# Interactions of Truncated Menaquinones in Lipid Monolayers and Bilayers

**DOI:** 10.3390/ijms22189755

**Published:** 2021-09-09

**Authors:** Cameron Van Cleave, Jordan T. Koehn, Caroline Simões Pereira, Allison A. Haase, Benjamin J. Peters, Seth W. Croslow, Kyle G. McLaughlin, Katarina R. Werst, Audra L. Goach, Dean C. Crick, Guilherme Menegon Arantes, Debbie C. Crans

**Affiliations:** 1 Department of Chemistry, Colorado State University, Fort Collins, CO 80523, USA; camvc@colostate.edu (C.V.C.); jordan_koehn@med.unc.edu (J.T.K.); Allison.Haase@colostate.edu (A.A.H.); ben.peters252@gmail.com (B.J.P.); katarina.werst@gmail.com (K.R.W.); 2 Department of Biochemistry, Institutio de Química, Universidade de São Paulo, Av. Prof. Lineu Prestes 748, São Paulo 05508-900, SP, Brazil; caroline013@gmail.com (C.S.P.); garantes@iq.usp.br (G.M.A.); 3 Department of Chemistry, Monmouth College, Monmouth, IL 61462, USA; SCROSLOW@monmouthcollege.edu (S.W.C.); kmclaughlin@mcw.edu (K.G.M.); ASOSTARECZ@monmouthcollege.edu (A.L.G.); 4 Cell and Molecular Biology Program, Colorado State University, Fort Collins, CO 80523, USA; dean.crick@colostate.edu; 5 Department of Microbiology, Immunology and Pathology, Colorado State University, Fort Collins, CO 80523, USA

**Keywords:** Menaquinone (MK), Vitamin K_2_, lipoquinone, phospholipid, Langmuir monolayer, bilayer, conformation

## Abstract

Menaquinones (MK) are hydrophobic molecules that consist of a naphthoquinone headgroup and a repeating isoprenyl side chain and are cofactors used in bacterial electron transport systems to generate cellular energy. We have previously demonstrated that the folded conformation of truncated MK homologues, MK-1 and MK-2, in both solution and reverse micelle microemulsions depended on environment. There is little information on how MKs associate with phospholipids in a model membrane system and how MKs affect phospholipid organization. In this manuscript, we used a combination of Langmuir monolayer studies and molecular dynamics (MD) simulations to probe these questions on truncated MK homologues, MK-1 through MK-4 within a model membrane. We observed that truncated MKs reside farther away from the interfacial water than ubiquinones are are located closer to the phospholipid tails. We also observed that phospholipid packing does not change at physiological pressure in the presence of truncated MKs, though a difference in phospholipid packing has been observed in the presence of ubiquinones. We found through MD simulations that for truncated MKs, the folded conformation varied, but MKs location and association with the bilayer remained unchanged at physiological conditions regardless of side chain length. Combined, this manuscript provides fundamental information, both experimental and computational, on the location, association, and conformation of truncated MK homologues in model membrane environments relevant to bacterial energy production.

## 1. Introduction

**Figure 1:**
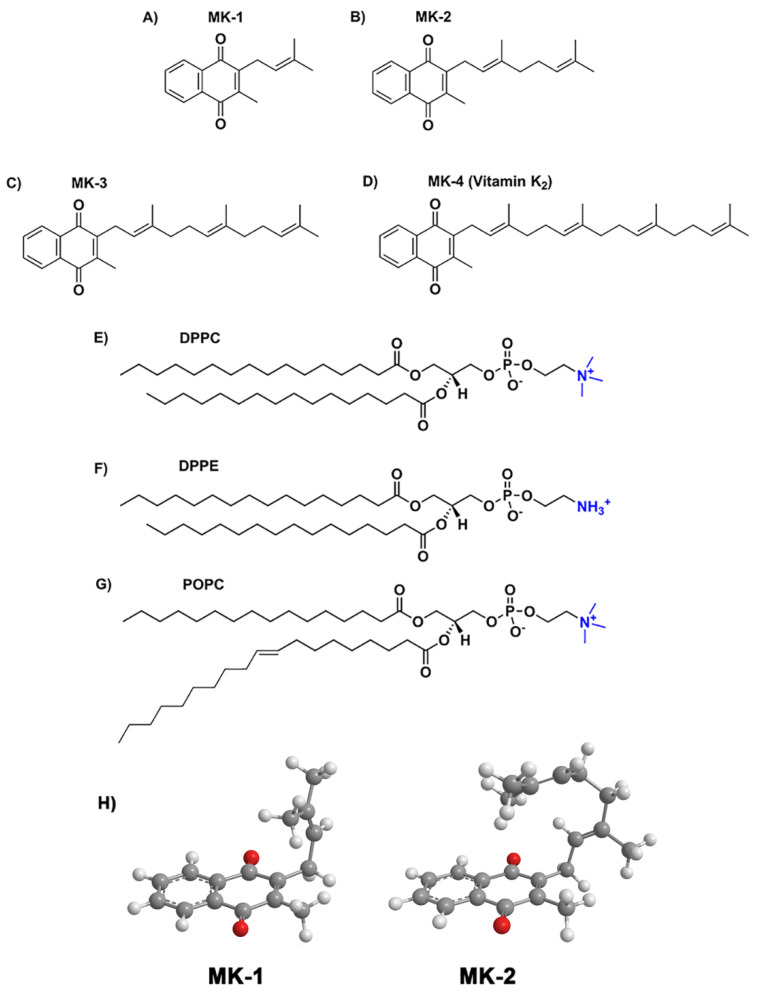
The structures of MK-1 through MK-4 (**A**–**D**) and the phospholipids (**E**) dipalmitoylphosphatidylcholine (DPPC), (**F**) dipalmitoylphosphatidylethanolamine, and (**G**) palmitoyloleyoylphosphatidylcholine (POPC). Conformations of (**H**) MK-1 and MK-2 within the AOT reverse micelle interface are adapted from refs [6,7].

Menaquinones (MK) belong to a class of molecules known as lipoquinones or lipid-quinones. MKs are cofactors used in the electron transport system (ETS) of bacteria to generate cellular energy, such as in the pathogenic *Mycobacterium tuberculosis*. [1,2,3] MKs consist of a naphthoquinone headgroup and an isoprenoid side chain of varying length (Figure 1) [4,5]. We have previously found that MK’s structure allows it to fold into different molecular shapes depending on environment and side chain length [6,7]. MKs must be membrane-associated to function in the ETS [1,8], and current knowledge regarding the interaction and conformation of MK homologues in phospholipid bilayers is limited and often conflicting [9,10,11]. Thus, understanding MK’s location, association, and conformation with membranes will ultimately provide a better understanding of bacterial energy production, which aids drug development to address the looming antibiotic resistance crisis [12,13,14,15]. MK homologues presumably reside in the hydrophobic region of the bilayer membrane due to the hydrophobicity of the MKs, and we are seeking experimental confirmation further specifying the location of truncated MKs. Studies in model membrane systems of the structurally similar lipoquinone analogue, ubiquinone (UQ), have been used successfully to determine that UQ is located near the water interface of the membrane [10,16,17], though there is some debate about whether the side chain is folded or extended. We anticipate that employing a similar methodology will enable us to characterize the behavior of MK homologues within membranes. In this manuscript, we use a combination of experimental and computational methods to investigate the location, association, and conformation of a series of MK homologues with varying isoprenyl side chain length (MK-1, MK-2, MK-3, MK-4) in the membrane. We used shorter MK homologues in our studies because they are less hydrophobic, which enables their study in aqueous-based systems, such as enzyme assays [3,18,19].

Langmuir monolayers are a model membrane system that provides information on packing, disruption, and location of a target molecule in the context of a phospholipid monolayer. Langmuir monolayers consist of a single layer at the air-water interface, usually comprised of amphiphilic phospholipids or other lipid-like molecules [20,21]. In this study, we used the phospholipids dipalmitoylphosphatidylcholine (16:0 PC, DPPC) and dipalmitoylphosphatidylethanolamine (16:0 PE, DPPE), which were mixed with the hydrophobic MK homologues to form a monolayer film [22]. Previous Langmuir monolayer studies have been performed with different UQ homologues. These UQ homologues were found to expand and disrupt the packing of the phospholipids as the length of the UQ isoprenoid side chain increased until approximately physiological surface pressure (30–35 mN/m) [23], when the UQ molecules were compressed into the hydrophobic phospholipid tails [24,25,26]. We expect to see a similar trend with the truncated MK homologues. However, since MKs are more hydrophobic than UQ, MKs may prefer to reside farther into the phospholipid tails at lower surface pressures.

We used molecular dynamics (MD) studies to provide support for the Langmuir monolayer experimental studies. Furthermore, MD simulations were used to obtain a more in-depth molecular view of the location, association, and conformational folding of the MK homologues in a simulated bilayer membrane system. In this manuscript, we used a previously validated MD bilayer system consisting of POPC with a single MK molecule in each membrane leaflet, which corresponds to an approximately 3% concentration of MK in the phospholipid bilayer [27,28]. This is a more physiologically relevant system than what we used in the Langmuir monolayer studies, but nonetheless complements the DPPC and DPPE Langmuir monolayer studies. Previous MD simulations with UQ placed the headgroup near the membrane interface by the phosphate group of the phospholipid with the isoprenoid side chain extended into the middle of the bilayer [27]. We hypothesize that under physiological conditions, (i) the hydrophobic MK headgroup will be located further away from the interfacial water than UQ, (ii) the side chain length influences the association of MKs with phospholipids, and (iii) the MK homologues adopt some type of folded conformation in a membrane environment.

## 2. Results

### 2.1. Compression Isotherm Studies of MKs in Langmuir Monolayers

Compression isotherms of Langmuir monolayers were obtained to provide insight into the interactions between the truncated MK-1 through MK-4 homologues with both DPPC or DPPE phospholipids. Langmuir monolayers are often used to examine the ability of a molecule to penetrate an interface, to disrupt packing, and to affect the elasticity of the monolayer [7,29,30,31]. We have previously reported compression isotherms of mixed films in terms of area per molecule for MK-1 and MK-2 [6,7]. The Langmuir monolayer data may be analyzed differently depending on the system of interest (hydrophilic vs. hydrophobic target molecule). Here, we normalized to the area per phospholipid because that allows for more facile interpretation of the results and comparison between multiple compounds such as MK-1, MK-2, MK-3, and MK-4. A similar analysis was previously used by Quinn and Esfahani in 1980 [32].

**Figure 2:**
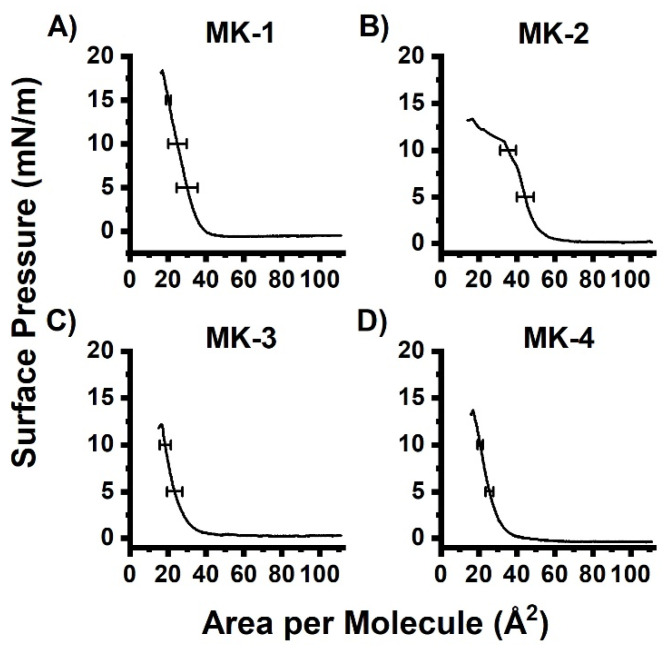
Compression isotherms of pure films of (**A**) MK-1, (**B**) MK-2, (**C**) MK-3, and (**D**) MK-4. Curves are the average of triplicate measurements. Error bars represent the standard deviation of the area.

The pure MK-2 monolayer reached a maximum pressure of ~13 mN/m (Figure 2B). This result is slightly lower than previously reported (20 mN/m) [7]. As found in previous studies, target MK homologues can undergo varying degrees of self-aggregation and are likely to cause small differences reported between MK experiments [7]. The pure MK-3 monolayer collapsed at 12 mN/m and the pure MK-4 monolayer reached a maximum pressure of 13 mN/m. A potential decrease in collapse pressure of these MK homologues as the isoprene side chain length increased was experimentally indistinguishable in contrast to the larger differences reported with ubiquinones [32].

We sought verification that a film was formed because the surface pressure does not begin to rise until ~40 Å^2^/molecule. Hysteresis studies were therefore performed on pure MK films to determine film stability (See Supplemental Information). All truncated MK homologue films showed a decreased surface area with each compression cycle, which confirmed the formation of films (Figure S2a). The decreased surface area demonstrates that MK films are all unstable and inelastic. A decrease in observed surface area may indicate that MKs are either self-aggregating or dissolving into the aqueous subphase. We anticipated that the most soluble MKs would form the least stable films due to the compound continually dissolving into the subphase. In hysteresis studies. the most elastic films are those films which are able to compress and expand multiple times and remain the same, such as the most stable film. We would have anticipated that MK-1 and MK-2 formed less elastic films due to their ability to dissolve into the subphase. However, even though MK-1 and MK-2 are more soluble in aqueous solutions, and they formed more elastic films. Both MK-3 and MK-4 form less stable films, which implies that self-aggregation is a more important contributor to film inelasticity than solubility.

**Figure 3:**
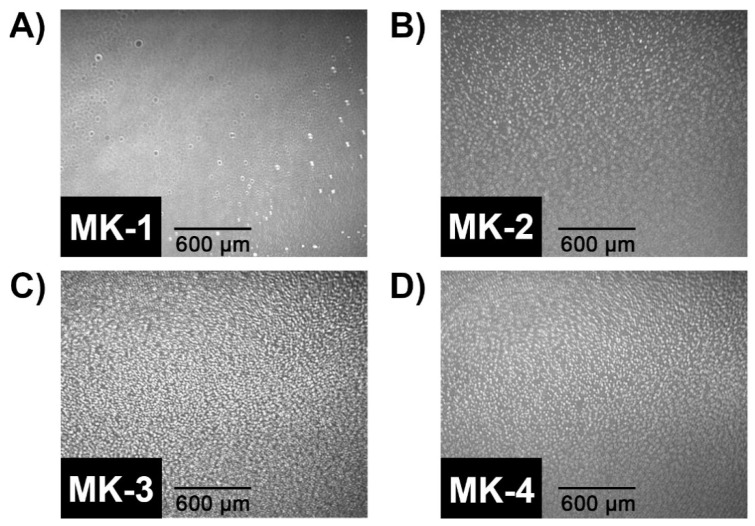
BAM images of pure MK films that demonstrated droplet-like structures were formed between 7.5 and 13 mN/m of surface pressure during compression. Images are shown at the approximate collapse pressure of each MK homologue. Images of (**A**) MK-1 (800 nmol), (**B**) MK-2 (120 nmol), (**C**) MK-3 (80 nmol), and (**D**) MK-4 (80 nmol) were captured at 12.5 mN/m, 10.0 mN/m, 11.8 mN/m, and 10.7 mN/m, respectively. Each panel is 2387 × 1925 µm. Images in this figure were cropped from raw images (640 × 480 px) to a final size of 382 × 308 px. All images were cropped from the upper right corner for consistency. Cropped images were then scaled up to 720 × 582 px. All image manipulation was performed in GIMP 2.10.22.

We obtained BAM images of MK homologues in order to obtain visualize the surface morphology of MK films, as shown in Figure 3. At the start of compression, a gray surface was observed, which indicates no organization. Upon reaching pressures >7 mN/m (collapse point in Figure 2), white circular features were observed, which indicates aggregation. In Figure 3A, we show a BAM image captured of MK-1, documenting that some aggregation occurred. Ten times the amount of MK-1 relative to MK-3 and MK-4 was needed to obtain meaningful BAM images. This may be due to MK-1 dissolving into the aqueous subphase [6]. Images of MK-2, MK-3, and MK-4 demonstrate that the surface was densely covered with MK aggregates. It is clear from these images that MK-1 behaves differently from the other three MK homologues.

**Figure 4:**
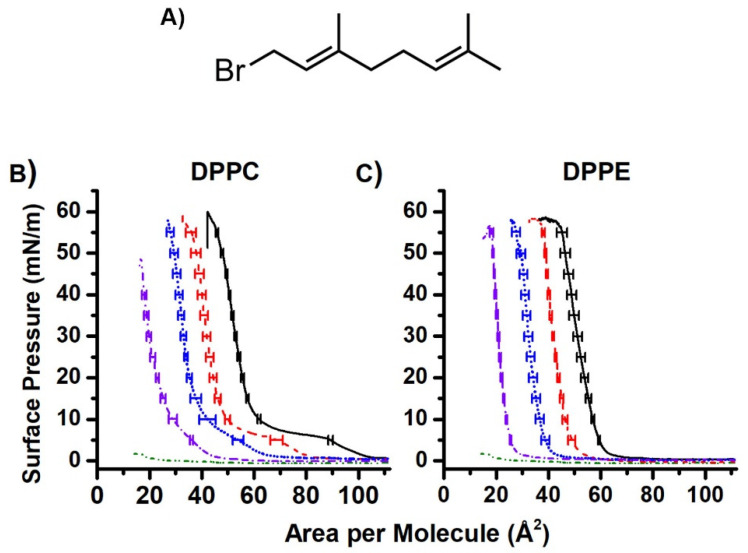
Compression isotherms of mixed films containing geranyl bromide. (**A**) The structure of geranyl bromide. (**B**) Compression isotherms of geranyl bromide and DPPC. (**C**) Compression isotherms of geranyl bromide with DPPE. Red dashed curves show 75:25 phospholipid:geranyl bromide films. Blue dotted curves are 50:50 phospholipid:geranyl bromide films. Purple dash-dot curves are 25:75 phospholipid:geranyl bromide films. Green dash-dot-dot curves are pure geranyl bromide films.

Geranyl bromide (*trans*-1-bromo-3,7-dimethyl-2.6-octadiene, Figure 4A) was used to further investigate a surface inactive compound that shares structurally similarity to MK. Geranyl bromide is a relatively surface-inactive molecule that contains a two-unit isoprenoid chain and a bromine atom in place of a headgroup, which provides an appropriate comparison for MKs. The related farnesol (containing three isoprene units) and farnesyl diphosphate have been reported to favor extended conformations in a number of solvents and in X-ray structures coordinated to proteins [33]. When applying geranyl bromide to the air-water interface and then compressing, the surface pressure remained at 0 mN/m until the end of compression when it rose to ~3 mN/m (Figure 4B,C). The surface pressure of geranyl bromide was significantly lower than the pure truncated MK films (10–17 mN/m). Given this information, MKs are surface-active but are unable to form a stable, elastic film. These compression isotherm and hysteresis studies confirmed that MK-1 through MK-4 form films, but the films are inelastic. In contrast, geranyl bromide, which lacks a headgroup, did not form a film.

At high geranyl bromide concentrations above 50% mol fraction, a disappearance of the gas-liquid transition in DPPC was observed (0–6 mN/m). While geranyl bromide is relatively surface inactive, it is likely affecting the packing of the model membrane at low surface pressure, but not at physiological pressure.

### 2.2. Compression Isotherms of Normalized Mixed MK and DPPC or DPPE Films

**Figure 5:**
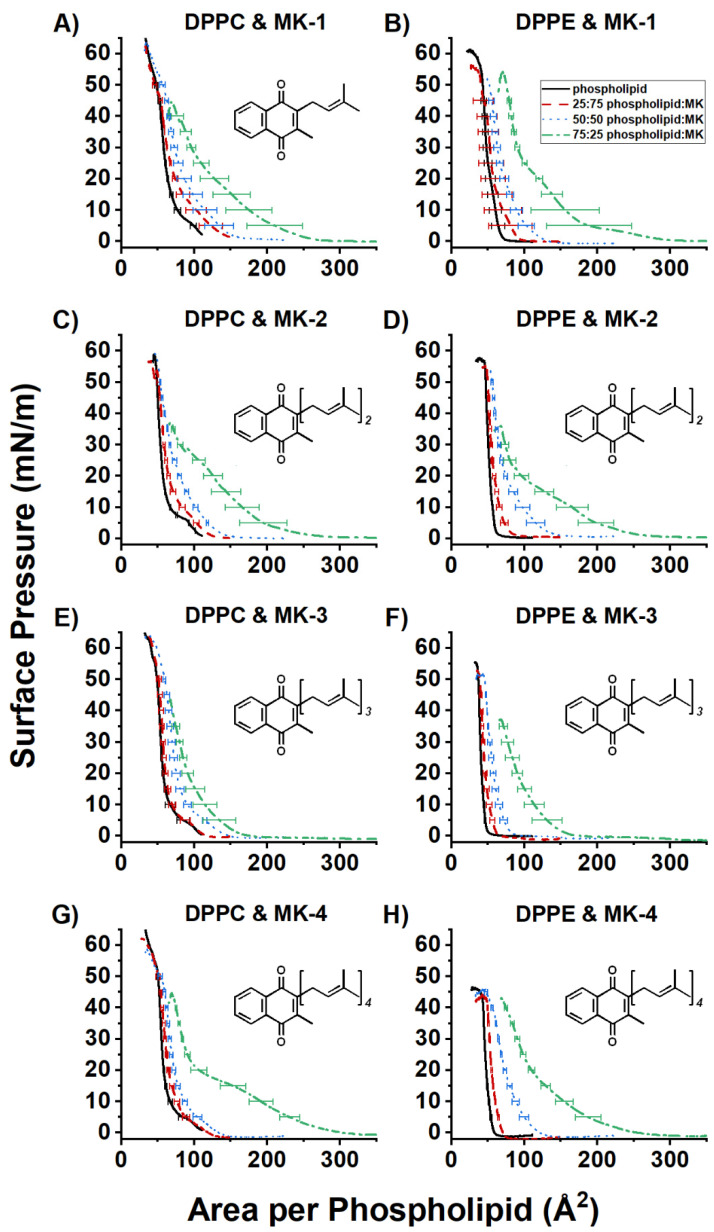
Normalized compression isotherms of mixed monolayers of either DPPC (left column) or DPPE (right column) with MK. Panels (**A**) and (**B**) are MK-1, (**C**) and (**D**) are MK-2, (**E**) and (**F**) are MK-3, and (**G**) and (**H**) are MK-4. Pure phospholipid monolayers are represented with solid black curves, 75:25 phospholipid:MK with red dashed curves, 50:50 phospholipid:MK with blue dotted curves, and 25:75 phospholipid:MK with green dash-dot curves. Each curve is the average of at least three replicates. Error bars are the standard deviation at every 5 mN/m of surface pressure. Data for MK-1 and MK-2 were previously reported [6,7].

The compression isotherms were measured for MK-1, MK-2, MK-3, and MK-4 and the normalized compression isotherm curves for the mixed monolayers of MK-1, M-2, MK-3, and MK-4 were replotted as a function of area per phospholipid, Figure 5. Normalization occurs by using Equation (1) where *A_N_* is the normalized area per phospholipid (Å^2^), *A* is the measured area per molecule (Å^2^), and *x* is the molar fraction of phospholipid (either 0, 0.25, 0.5, 0.75, or 1).

(1) $$A_{N}=A\left(x^{-1}\right).$$

Mixed films of MK-1 and DPPC show an overall increase in area as the molar fraction of MK-1 increases, though the 75:25 and 50:50 phospholipid:MK-1 curves are similar (Figure 5A). The typical gas-liquid transition (0–6 mN/m) seen in the pure DPPC curve disappears in the mixed monolayers. In addition, the 25:75 DPPC:MK-1 film did not undergo a full collapse (end of compression where there is no longer a monolayer). This trend is also seen with mixed film containing MK-2, MK-3, and MK-4. In addition, increasing amounts of MK were found to increase the compressibility of both DPPC and DPPE mixed monolayers by compression modulus analysis (see Supplemental Information).

Studies with DPPE are more difficult to interpret because there is only one phase change. Therefore, we will only focus on large differences observed between the data. Overall, gentler slopes were observed with increasing molar fractions of all MK molecules with DPPE. However, the 50:50 and 75:25 DPPE:MK-4 films exhibited a liquid condensed phase from 1 mN/m to 17 mN/m. The liquid condensed phases seen in the DPPE:MK-2 and DPPE:MK-4 mixed films indicate an expansive effect, which is observed in literature with UQ [34,35]. This expansion at lower surface pressures may be due to aggregation and/or conformation of the MK homologues. Interestingly, for both DPPC and DPPE, the mixed curves tended to overlap the control curve at physiological surface pressure (30–35 mN/m) [23]. This has previously been observed with UQ and was interpreted as the lipoquinone migrating out of the interface and into the phospholipid tails [24]. These studies confirm the interpretation that MK homologues reside slightly higher in the interface than UQ, thus confirming our initial hypothesis that MK and UQ reside in differing locations in model membranes.

### 2.3. Ideal Mixing of MK and DPPC or DPPE

**Figure 6:**
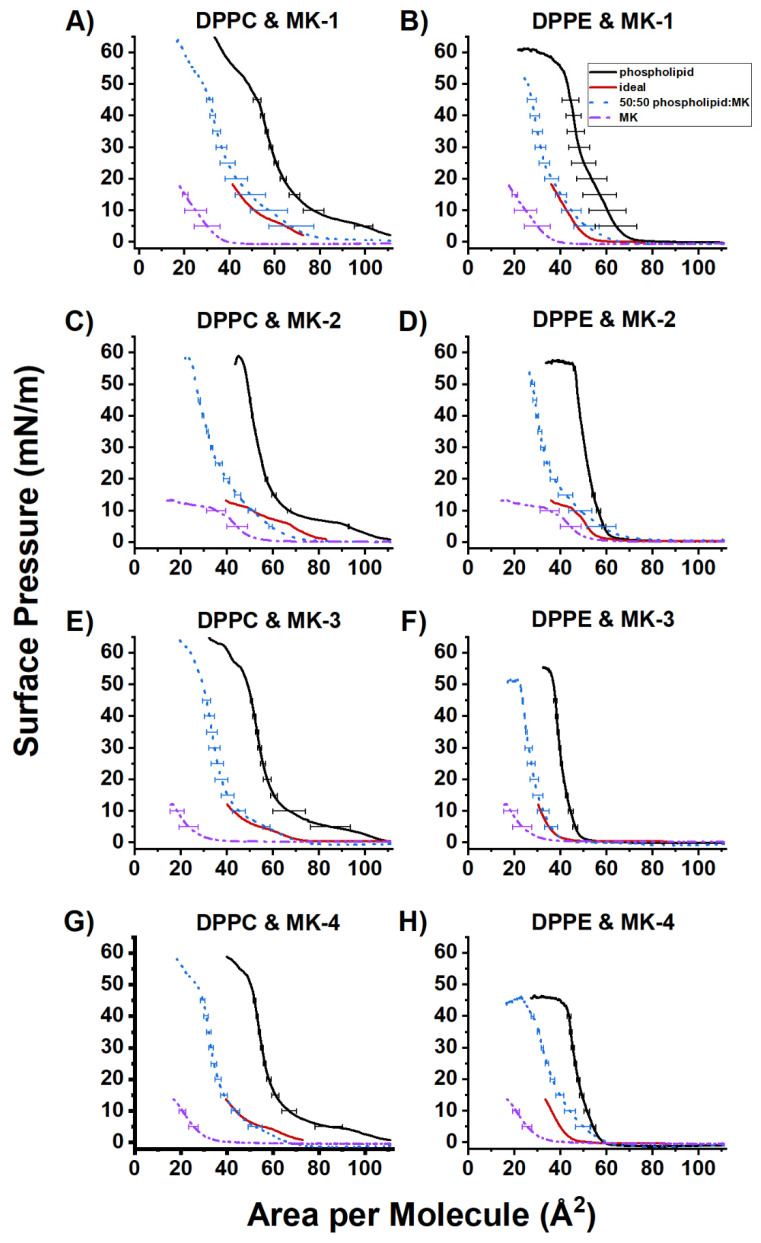
Ideal mixing of 50:50 phospholipid:MK films compared to experimental data. DPPC films are in the left column. DPPE films are in the right column. (**A**,**B**) show MK-1 mixed films, (**C**,**D**) show MK-2 mixed films, (**E**,**F**) show MK-3 mixed films, and (**G**,**H**) show MK-4 films. Solid black curves are pure phospholipid monolayers. Blue dotted curves represent experimental 50:50 phospholipid:MK films. Solid red curves represent calculated ideal mixed films. Purple dash-dot-dot curves represent pure MK films. Each MK homologue experiments were run independently, hence differences in control isotherms were observed.

Ideal mixing calculations were performed to confirm whether or not any interactions were occurring between phospholipids and MK homologues, as well as the differences in the free energy of the films, as shown in Figure 6. The ideal mixing was plotted to show where the ideal and experimental fall relative to both the MK and phospholipid control (plotted using un-normalized data). Assuming no interactions between the two components of the film, the experimental film will match the calculated ideal. Ideal mixing curves for 50:50 phospholipid:MK mixtures are presented in the main text as representative results while curves for 25:75 and 75:25 phospholipid:MK mixtures are shown in Supplemental Information (Figure S5a,b).

The general trend of the 50:50 DPPC:MK films indicate ideal mixing, in that the experimental curves do not deviate significantly from the ideal. As such, DPPC and the MK homologues likely do not interact directly with each other. In the DPPE films, the 50:50 mixture containing MK-4 is expanded relative to the ideal mixing area. This suggests that MK-4 is able to associate with DPPE, possibly due to conformational folding and molecular shape. We sought further means of computationally investigating molecular reasoning for this, specifically MD simulations.

Langmuir monolayers studies were studied at both low and high molar fractions that were well above the biological molar fraction to observe the association of MK homologues on the DPPC and DPPE films [9]. As such, it is not clear whether the observed effects at higher molar fractions in the monolayers are relevant to effects observed within bilayers and native membranes. We hypothesized that conformation might be important for the disruptive association of MK homologues at high molar fractions. However, Langmuir monolayer studies were unable to provide molecular information on folding and the exact mechanism of disruption between phospholipids and MK homologues. As such, we investigated this question using a computational model to probe the MK conformation in a physiologically relevant bilayer system.

### 2.4. Molecular Dynamics Simulations of MKs in a Membrane Bilayer

**Figure 7:**
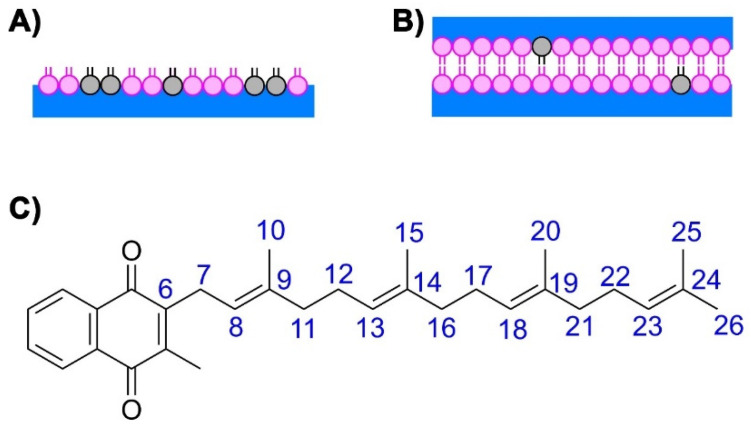
Cartoons of different model membrane systems as well as the numbering of carbons for computational studies. (**A**) Illustration of the monolayer system with a mix of phospholipid (pink) and MK (grey). (**B**) Illustration of a bilayer system with a molecule of MK in each of the phospholipid layer in the bilayer, (**C**) Labeling scheme of MK-1 through MK-4 (MK composed by *n* = [1–4] isoprenoid units) used in computational studies. The terminal carbon (CT) groups on MK-1 are labeled 10 and 11, the CT groups on MK-2 are labeled 15 and 16, the CT groups on MK-3 are labeled 20 and 21, the CT groups on MK-4 are 25 and 26.

Computational studies were performed to determine the location, association, and conformation of MK homologues embedded in a bilayer at physiological concentrations. We modeled fully hydrated bilayers based on the phospholipid phosphatidylcholine (POPC, 16:0x2013;18:1 PC) mixed with one MK molecule in each layer, which correspond to a ~2–3% concentration of MK-1, MK-2, MK-3, or MK-4 (Figure 7A). Classical MD simulations were performed with the CHARMM36 force field using parameters for MKs developed previously [27,28].

The Langmuir monolayer studies showed that at lower MK concentrations (25% molar fraction), the MK homologues were associated with the monolayer film. However, at higher concentrations the MK homologues were compressed out of the film (Figure 5). In the computational studies with the phospholipid bilayer, in no example was the MK homologue compressed out from the phospholipid bilayer at physiological conditions. The lack of MK exclusion from the bilayer is likely due to two reasons: (i) lower MK concentrations similar to those existing under biological conditions were investigated and (ii) a finite simulation time (350–750 ns) was used, which may not be enough time to sample the water-phospholipid partition process [9].

**Figure 8:**
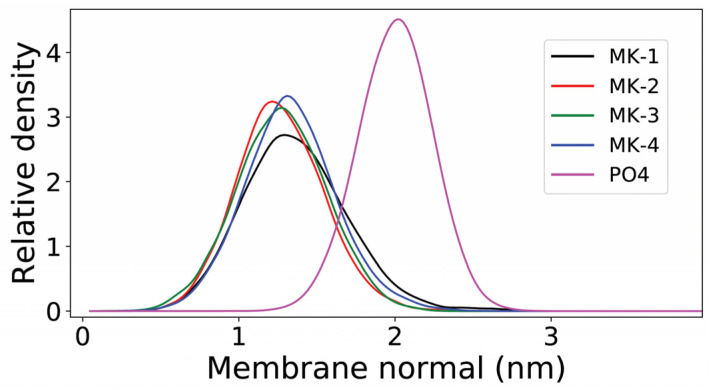
Mass density of the MK quinone headgroup along the membrane normal for MK-1 (black), MK-2 (red), MK-3 (green), and MK-4 (blue). The phosphate group of POPC (PO4) is shown in magenta. Data from both layers were symmetrized. The normal zero corresponds to the center of the bilayer.

Figure 8 details the position of the MK headgroup in the bilayer in terms of center of mass. The plot shows the distance from the center of the membrane (0 nm) and the interface as indicated by the POPC phosphate group’s center of mass at about 2 nm. As shown in the plot for MK-1, MK-2, MK-3, and MK4, the center of mass for the MK headgroups were all located around z = 1.3 nm. The small variations in peak position are not statistically significant. Thus, the MK headgroups are about 0.7 nm up in the interface and below the water-phospholipid interface as defined by the phospholipid phosphate (2 nm). Our simulations show that the MK headgroups will have the same location in the membrane, regardless of difference in hydrophobicity, length of the MK side chain, and ability to disrupt the membrane. These studies are in line with previous simulations of native UQ in POPC and mixed membranes [27,28], and suggest that these lipoquinone headgroups are both located in a similar membrane region, about 0.5 nm below UQ (z = 1.8 nm) toward the membrane midplane. These data also support the interpretation that for truncated MK homologues, the headgroup anchors the location of the MK homologue slightly farther into the membrane than that of UQ (MK z = 1.3 nm, UQ z = 1.8 nm, POPC phosphate z = 2 nm) [27,28]. The location of MK in a more hydrophobic region compared to UQ is consistent with Langmuir monolayer findings that placed the MK homologues in the phospholipid tails at physiological surface pressure. In addition, there was no appreciable disruption to the permeability of the bilayer noted in simulations which is in agreement with previous studies [27,28].

**Figure 9:**
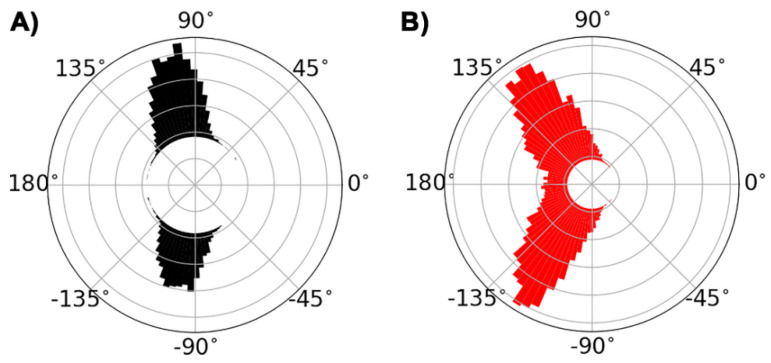
Polar plot showing distributions of dihedral angles rotating around the C6-C7 bond (panel (**A**)) and the C7-C8 bond (panel (**B**)) observed in the MD simulation of MK-4 located in the phospholipid POPC bilayer. Interestingly, the energy function observed when rotating around the C6-C7 bond is not symmetrical because the molecular shape is not symmetrical. Steric repulsions to the naphthoquinone ring substitutions are directional as described previously in detail [27].

The MD studies provided a quantitative representation of the conformation and distribution of the dihedral angles of the side chain of MK-1 through MK-4 in a simulated phospholipid bilayer (Figure 9). Rotation around the C6-C7 bond was restrained in all MK homologues due to the steric restriction that limits rotation. Specifically, the methyl group on the naphthoquinone headgroup and the sp^2^ hybridization of the C6 atom limit the rotation around the C6-C7 bond. Thus, this torsional angle is ±110° (Figure 9A). Rotation around the C7-C8 bond was freer than around the C6-C7 bond but still somewhat restrained due to the methyl group on the naphthoquinone headgroup and the sp^2^ hybridization of C8. The bond angle was often ±120°, but some trans (180°, extended) conformations were also present in Figure 9B.

**Figure 10:**
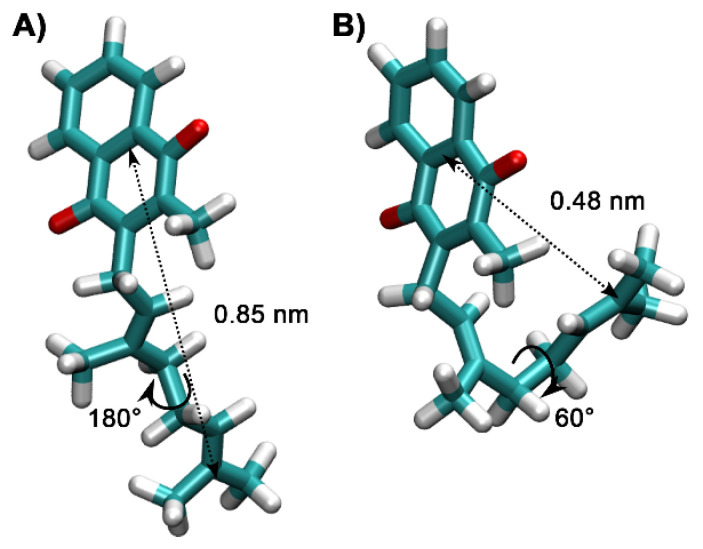
Two representative conformers observed for the MK-2 MD simulations in the POPC bilayer. Torsion around the C11-C12 bond modulates the distance between the terminal CH_3_-carbon labeled CT and the center of the quinone ring, termed here d(CT-H). Panel (**A**) shows a trans conformer with a long intramolecular distance and panel (**B**), a gauche conformer with a much smaller intramolecular distance.

Torsional angle distributions of corresponding rotations around C6-C7 and C7-C8 bonds similar to Figure 9, were observed for all MK homologues studied here. However, MK-2, MK3, and MK-4 contain longer side chains and additional C-C bonds, which are more flexible than MK-1. Figure 10A shows a trans (extended) conformer in which the C11-C12 torsional angle is ±180°. Figure 10B shows that the gauche (folded) conformer (C11-C12 torsion is ±60°) will allow for partial folding of the side chain over the naphthoquinone headgroup. Overall, truncated MK homologues undergo some amount of folding in a phospholipid bilayer.

**Figure 11:**
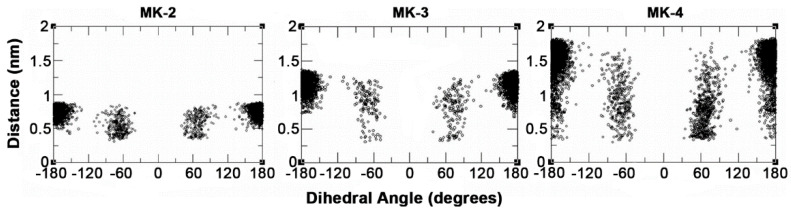
Plots of the distance between the terminal CH_3_ groups in MK-2, MK-3 or MK-4 to the middle of the center C-C bond of the naphthoquinone headgroup, d(CT-H), as obtained when the dihedral angle is changing as the rotation around the C11-C12 bond takes place.

Figure 11 shows the distances (termed d(CT-H)) generated through rotation of the dihedral angle (rotation around the C11-C12 bond) between the terminal CH_3_ group (CT) and the C2-C3 (UQ numbering) bond in the middle of the naphthoquinone headgroup; the different distances are observed due to rotations around the C11-C12 bond. The panels in Figure 11 all show conformations with angles in *trans* (~180°, extended) more populated than the conformation with *gauche* (±60°, folded) geometry for MK-2, MK-3, and MK-4. However as shown in Figure 11 for MK-3, the relative population of *gauche* is significantly lower than for MK-2 or MK-4.

**Figure 12:**
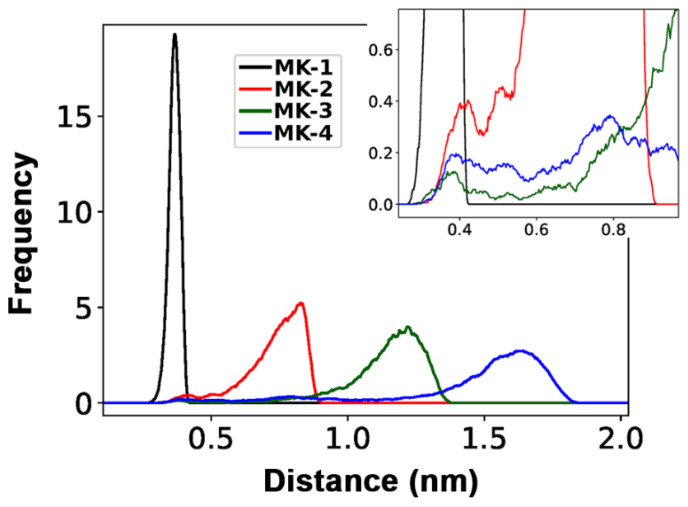
Distance distribution from terminal carbon (CT) to the center of the MK quinone headgroup, d(CT-H) in MK-1 (black), MK-2 (red), MK-3 (green), and MK-4 (blue). The upper right panel is a zoom-in of the distance range 0.3–1.0 nm (3–10 Å).

Figure 12 summarizes the population distribution of the terminal carbon from the C-C bond in the middle of the headgroup for all MKs. As the isoprenoid side chain length increases, there is the potential for a greater distance between the terminal carbon and the headgroup. Since MK-1 has limited length and rotation, the entire distribution occurs within a small range of distances. MK-2 can reach d(CT-H) <0.5 nm only when C11-C12 is in gauche conformation. In the case of MK-3 and MK-4, short distances can be reached when C11-C12 in gauche as well as trans because their isoprene chains contain additional rotatable C-C bonds and are long enough to fold back over the headgroup. Figure 11 shows that d(CT-H) >0.7 nm when C11-C12 in MK-3 is trans. Even when in gauche, fewer MK-3 conformations will have a smaller d(CT-H). MK-4 may reach d(CT-H) <0.5 nm when C11-C12 is trans, because of increased side chain length and flexibility of the additional isoprenoid units. As shown in Figure 10, C11-C12 torsion in gauche allows the side chain to partially fold upon itself and, thus, a lower d(CT-H) to be visited. Similar results (data not shown) are obtained if we examine the equivalent torsions for bonds closer to the CT, such as the C16-C17 bond in MK-3 and MK-4.

## 3. Discussion

Langmuir monolayers were used to experimentally probe the location and association of MK homologues within phospholipid monolayers. There are two ways to conduct Langmuir monolayer experiments depending on the solubility of the compound of interest. When the compound is water-soluble, it is added to the aqueous subphase. With hydrophobic molecules, experiments are conducted by mixing and applying different molar ratios of substrate and phospholipid, as described by Hoyo et al. in 2015 [22]. In order to observe a response on the monolayer, concentrations of the target compound are typically higher than micromolar. This is above the solubility of even the water-soluble truncated MK homologues. In our studies using molar ratios of phospholipid vs. MKs, information about potential aggregation of MK homologues and film formation was gathered [36,37]. Using the Langmuir trough, we studied how truncated MKs (MK-1, MK-2, MK-3, and MK-4) associated with DPPC and DPPE films.

Biologically, DPPC is present in up to 40% of mammalian lung surfactant while little, if any, is found in bacterial membranes [36,38]. However, DPPC has been well characterized in Langmuir monolayers and demonstrates distinct behavioral phases (gas, gas-liquid, liquid condensed, solid) which give information on the disruption of phospholipid packing. Therefore, it is used extensively in model membrane systems [39]. DPPE is found in bacterial cells and is only a minor component in mammalian cells, such as in the inner leaflet of eukaryotic cells [40,41,42]. As such, DPPE is the most biologically relevant phospholipid for the study of MKs. While the more biologically plentiful POPC has been used in Langmuir monolayer studies, it did not demonstrate the same phase changes as DPPC and is therefore less informative with regards to the association of MK homologues [43]. Compression isotherms in this manuscript were accordingly measured at 25 °C to maintain the distinct phases of DPPC, as the gas-liquid phase is not present at physiological temperature [44].

We investigated the ability of truncated MK homologues to form films. We found that MKs were surface-active even though the surface pressure did not begin rising until ~40 Å^2^/molecule and that these MK films were unstable. Using BAM, we were able to visualize the aggregation of the MK homologues and we observed strong aggregation of MK-2, MK-3, and MK-4. However, the self-association with MK-1 was weaker, possibly to enhance water solubility. Because of the limiting solubility of the MK homologues, the studies of the MK derivatives on the films were performed using ratios of MK homologue to DPPC or DPPE. By mixing ratios of phospholipid and MK, we found that the MK homologues associated with the phospholipid interface, and that the at low surface pressure disruptive effects were greatest for MK-2 and MK-4, as shown in Figure 5. However, we observed little, if any, increase in disruptiveness between 30 and 35 mN/m. Moreover, the curves of 75:25 phospholipid:MK overlap the phospholipid control in all but the DPPE:MK-4 trials. The conclusions of the lack of disruption at physiological surface pressure are that (i) the MK homologues were compressed into the phospholipid tails from the interface and (ii) that this migration to the saturated phospholipid tails allows for greater accommodation of the volume of the MK homologues, hence the lack of disruption. We used MD simulations to confirm the location and association with phospholipids and additionally explore the conformation of MK homologues.

The MD simulations were performed in a phospholipid bilayer, and at a phospholipid:MK ratio that approximated the concentrations found in biological systems. We chose a model bilayer composed of MK homologues embedded in a POPC bilayer, which was previously developed in our laboratory to investigate the interaction with native UQs or MKs in eukaryotic cells [28]. Although simulations of Langmuir monolayers are possible [45], they would require an extensive reparametrization and testing of the force-field used for simulations [27,28] and would provide little detail on the biological context in which MKs are found. Instead, we chose to carry out simulations at a physiologically relevant MK concentration within a model phospholipid bilayer, which are more reliable with our current force-field technology [27,28], and resulted in detailed information on the intrinsic folding of MK isoprenoid chains in its (MK-4) native membrane environment. Eukaryotic membranes have a large POPC concentration and pure POPC bilayers have been well-characterized as models for the simulation of biological membranes [28,46]. In particular, we have previously characterized in detail the location and water-phospholipid partition ofUQ with variable isoprenoid chain length to POPC bilayers, in good agreement with experimental observations [27].

The MD simulations also show that once the MK was associated with the membrane, the average (equilibrium) location of the MK headgroup did not depend on the number of MK isoprenoid units (Figure 8). The tiny differences observed between the four MKs in Figure 8 are not statistically significant and are due to fluctuations of the finite sampling. Thus, our simulations do not show any dependence of MK headgroup location with side chain length, in line with previous simulations of UQ with various side chain lengths in POPC and in mixed membranes [27,28]. The MD studies also suggested that the location of lipoquinones along the membrane midplane is an intrinsic physicochemical property of the quinone molecule due at least in part to its amphiphilic character and more polar headgroup. This finding supports the possibility that in the monolayer system the MK headgroup location will not change with isoprenoid chain length. However, the redox state (quinone vs. quinol form) of the headgroup affects location, as we previously demonstrated within reverse micelle membrane environments [47]. Combined, our work supports the possibility that the headgroup structure and redox state, as opposed to side chain length, is a major contributing factor driving the location and association of MK homologues in a membrane.

The conformational distribution of C-C bonds in the MK side chain described in the MD bilayer simulation results have a subtle but potentially relevant impact on side chain folding upon the MK headgroup and the related distance d(CT-H) (Figure 9, Figure 10 and Figure 11). The possible distances for MK-1 are quite narrow due to rather restricted torsion around the C6-C7 bond (Figure 11). For the other MK, longer distances are reached and the distribution spreads due to increasing the number of isoprenoid units and increased side chain flexibility (Figure 11). The conformations where the side chain folds over the headgroup have a different shape compared to MK-1 where the side chain is at an angle with the headgroup. Interestingly, the exerpt in Figure 12 shows the terminal carbon of MK-3 is less likely to reside near the naphthoquinone headgroup than MK-2 or MK-4. Thus, we suggest that the non-ideal behavior observed for these MK homologues in the monolayer isotherms (Figure 6) may be caused by the more frequent partial folding of the side chain over the MK head group as observed in the MD simulations for MK-2 and MK-4 and the related shorter d(CT-H) (Figure 12).

The interactions of lipoquinones with membranes are a multi-faceted topic in which many different factors are important to the conformation and location of the lipoquinones in the phospholipid bilayer. In order to illustrate some of these effects, we compared the properties of the different MK homologues, as shown in Table 1. We ordered the properties of MK-1 through MK-4 in terms of clogP, ability to disrupt a monolayer (based on increase in monolayer area at physiological surface pressure), MK headgroup location, longest average distance of the terminal carbon of the isoprene chain from the naphthoquinone headgroup, and the ability of the terminal carbon to reside within 0.6 nm of the naphthoquinone headgroup (which is a measure of folding). The only two properties that showed the same order are the clogP and the longest average distance of the terminal carbon of the isoprene side chain from the naphthoquinone headgroup.

We confirmed that MK homologues occupy a more hydrophobic region of the membrane than UQ, though there was less disruption of phospholipid packing. We hypothesize that the lack of disruption is due to the location of the MK homologues. The free rotation of the phospholipid tails tails allows for compensation of the molecular volume of MK while UQ’s location close to the phospholipid headgroups in the interface does not [17,48]. In addition, we also found that all MK homologues adopted some folded conformation in a simulated bilayer, though conformations varied. We would be interested in exploring the physicochemical properties of the reduced quinol forms of these MK homologues. However, menaquinols are unstable under atmospheric conditions, making experimentation difficult [11,47].

## 4. Materials and Methods

**General Materials and Methods**. Chloroform (≥99.5%, monosodium phosphate (≥99.0%), disodium phosphate (≥99.0%), sodium hydroxide (≥98%), hydrochloric acid (37%), geranyl bromide (*trans*-1-bromo-3,7-dimethyl-2,6-octadiene, 95%), and manequinone-4 (MK-4, menatetrenone, Vitamin K_2_) were all purchased from Sigma Aldrich (St. Louis, MO, USA) and used without further purification. The phospholipids dipalmitoylphosphatidylcholine (16:0 PC, DPPC, 99%, SKU 850355P) and dipalmitoylphosphatidylethanolamine (16:0 PE, DPPE, 99%, SKU 850705P) were purchased from Avanti Polar Lipids (Alabaster, AL, USA) and pure lyophilized powder. Since MK-1, MK-2, and MK-3 are not commercially available, they were synthesized and purified as previously described [6,7,49]. Distilled deionized (DDI) water was filtered through a Millipore water purification system (Burlington, MA, USA) until water with a measured resistance of 18.3 MΩ was achieved. Langmuir monolayers were studied using a Kibron µTrough XS (stainless steel, Helsinki, Finland) equipped with a Teflon ribbon barrier.

**Preparation of Solutions**. The aqueous subphase of monolayers consisted of 20 mM sodium phosphate buffer (pH 7.40 ± 0.02).

Solutions were brought to pH with 1 M HCl or NaOH. Phospholipid stock solutions were prepared by dissolving DPPC (18 mg, 25 nmol) or DPPE (17 mg, 25 nmol) in 25 mL of 9:1 chloroform/methanol (*v*/*v*) for a final concentration of 1 mM phospholipid. MK and geranyl bromide stocks consisted of 1.0 mM MK-1 (12 mg, 5 nmol), MK-2 (15 mg, 5 nmol), MK-3 (19 mg, 5 nmol), MK-4 (22 mg, 5 nmol), or geranyl bromide (1.1 mg, 5 nmol) dissolved in 5 mL of 9:1 chloroform/methanol (*v*/*v*). Mixed phospholipid solutions were created by mixing appropriate amounts of phospholipid and either MK or geranyl bromide stock in a 2 mL glass vial to create a final volume of 1 mL and vortexed until combined. Final mol fractions (phospholipid:MK) were 100:0, 25:75, 50:50, 75:25, or 0:100.

**Preparation of Langmuir Monolayers**. The buffered aqueous subphase consisted of 50 mL of 20 mM sodium phosphate buffer (pH 7.40 ± 0.02) in DDI water (18.2 MΩ). The subphase surface was cleaned via vacuum aspiration until a quick compression of the subphase provided a surface pressure which was consistently 0.0 ± 0.5 mN/m throughout compression. A total of 20 μL of phospholipid stock solution (20 nmol of phospholipid) was then added to the surface of the subphase in a dropwise manner using a 50 μL Hamilton syringe. The monolayer was allowed to equilibrate for 15 min.

**Compression Isotherm Measurements of Langmuir Monolayers**. The phospholipid monolayer was compressed from two sides with a total speed of 10 mm/min (5 mm/min from opposite sides). The temperature was maintained at 25 °C using an external water circulator. The stainless-steel trough plate was scrubbed three times with isopropanol, then three times with ethanol, then rinsed with DDI water (18.2 MΩ) before each experiment. The Teflon ribbon barrier was rinsed with isopropanol followed by ethanol and then DDI water. The surface tension was monitored via Wilhemy plate technique where a steel wire was used as the probe instead of a metal or paper plate. The surface pressure was calculated from the surface tension using Equation (2), where *π* is the surface pressure (mN/m), *γ_0_* is the surface tension of water (71.99 mN/m), and *γ* is the surface tension at a given area per phospholipid after the monolayer has been applied.

(2) $$\pi =\gamma_{0}-\gamma .$$

Each compression isotherm experiment consisted of at least three replicates. The averages of the area per phospholipid and the standard deviation at every 5 mN/m were calculated using Microsoft Excel (=AVERAGE, =STDEV). The worked-up data were then transferred to Origin 2021 (Northampton, MA USA) to be graphed with error bars.

**Ideal Mixing of Monolayers**. The ideal mixing sets were calculated by averaging the mean molecular area of two isotherms at the same surface pressure using Equation (3), where *A_i_* is the ideal mixed area (Å^2^), *x_MK_* is the molar fraction of MK, *A_MK_* is the area per molecule (Å^2^) of the control MK monolayer, *x_PL_* is the molar fraction of DPPC or DPPE, and *A_PL_* is the area per molecule (Å^2^) of DPPC or DPPE. The possible mol fractions were 0.25, 0.50, or 0.75.

(3) $$A_{i}=x_{MK}A_{MK}+x_{PL}A_{PL}.$$

**Brewster Angle Microscopy.** Brewster angle microscopy (BAM) images were obtained using a Biolin NIMA medium trough (Gothenberg, Sweden) equipped with a MicroBAM (659 nm laser). Differing amounts of 2 mM stocks of MK-1 (800 nmol), MK-2 (120 nmol), MK-3 (80 nmol), and MK-4 (80 nmol) were added.

**Molecular Dynamics Simulations**. We employed a previously developed fully hydrated POPC (16:0-18:1 phosphatidylcholine) bilayer model system and added MK molecules (one MK in each layer, corresponding to a ~2–3% concentration), composed by *n* = [1–4] isoprenoid units (MK-1 through MK-4, Figure 1A and Figure 4D). Symmetric phospholipid bilayers were built containing 126 molecules of POPC and 7794 water molecules which have previously been characterized to represent a biological membrane [27]. NaCl was added until a final concentration of 150 mM was achieved. The protocol described by Javanainen was used to insert one MK-4 in each layer of the membrane [50]. The system was relaxed by a 50 ns MD run, and mean area and bilayer thickness were monitored to check for equilibration. Initial equilibrated configurations were derived from the MK-4 system by deleting side chain atoms and adapting the atomic connectivity to generate MK-1, MK-2, and MK-3 species. Conformations were sampled using classical MD simulations with the program GROMACS version 2020.3 [51] and the CHARMM36 force-field [52,53]. Parameters for MK were obtained by us previously [27,28]. Water was described by TIP3P [54] and the NPT ensemble was used. The temperature was kept at physiological temperature (37 °C, 310 K) with a Bussi thermostat [55] and a coupling constant of 0.1 ps. The pressure was kept at 1.0 bar with Parrinello-Rahman barostat for productive runs [56] with a coupling constant of 1 ps and a compressibility of 0.5 10^−5^ bar^−1^. Semi-isotropic coupling was applied. Electrostatic interactions were handled by Particle-Mesh Ewald (PME) [57] with grid spacing of 0.14 nm and quartic interpolation. All bonds were constrained using the LINCS algorithm [58]. No dispersion corrections were applied [59]. The integration time step was 2 fs and MD simulations were run 200 ns for equilibration. Trajectories with 350 ns were collected for MK-1, MK-2 and MK-3 and with 750 ns for MK-4.

## 5. Conclusions

MKs are membrane-associated lipoquinones that are used as essential components in the ETS of many bacteria. Therefore, understanding the behavior of MKs in membranes could provide fundamental knowledge of the ETS and could aid in antimicrobial drug development. We have previously demonstrated that truncated MKs fold in a model membrane interface. However, we sought more information on the location of MKs as well as how MKs associate with and affect the packing of phospholipids in a membrane environment. We hypothesized, but did not confirm, that MKs would behave in a similar manner to UQs, in that there would be a side chain-dependent disruption of phospholipid packing and association with MKs. Moreover, we wanted to further explore their predicted location and conformation in a membrane bilayer. We used a combination of experimental and computational methods to probe these open questions. Langmuir monolayer studies provided experimental data pertaining to phospholipid packing and association and MD simulations provided molecular information of exact location, association, and conformation in a membrane bilayer at physiological MK concentration.

Langmuir monolayers were created with biologically relevant phospholipids, DPPC and DPPE, to experimentally model the cell membrane interface. All truncated MK homologues were found to migrate from the air-water interface into the phospholipid tails at physiological surface pressure, which is consistent with our hypothesized location. We demonstrated that truncated MKs associate with the phospholipids but do not disrupt the phospholipid packing at physiological surface pressure that was observed with UQs [24,25,26]. We found using MD simulations that, in accordance with MKs hydrophobic nature, the MK headgroup was located closer to the phospholipid tails than UQ (UQ was located closer to the interfacial water) which is consistent with the hypothesized location. Furthermore, we found through MD simulations that MK-2, MK-3, and MK-4 overall favored a gauche, or folded, conformation, which is in agreement with our previous experimental studies with MK-1 and MK-2 [6,7]. In line with Langmuir monolayer studies, there was no observed dependence on MK side chain length for either MK conformation or location within the bilayer under physiological conditions. However, it is possible that this lack of dependence on MK side chain length is limited to truncated MK homologues and that the longer MK homologues, such as MK-9, would exhibit an appreciable difference in folding and disruption due to the significantly larger volume of MK-9. As the MKs are located further into the phospholipid tails than UQs, it is possible that the phospholipid tails adjust to compensate for the volume of the MK molecule. Hence, MK would be less disruptive than UQ based on membrane location.

Combined, Langmuir studies and MD simulations demonstrated that truncated MKs are located closer to the phospholipid tails, regardless of the truncated MK side chain length. A lack of dependence on side chain length was also observed in the association and packing of truncated MK homologues with phospholipids. Additionally, truncated MKs generally demonstrated some amount of folding. In conjunction with previous studies detailing the different, environment-dependent folded conformations of MK-1 and MK-2, this provides a fundamental view of the behavior of MKs in a membrane environment. Overall, MK homologues may disrupt phospholipid packing at higher concentrations as seen in Archaea [11,60], but not necessarily at concentrations found in most other organisms [9]. These truncated MK homologues were also found to adopt folded conformations, which may influence their behavior, recognition, and function in the ETS that is essential for bacterial survival.

## Figures and Tables

**Table 1:** Comparing various physicochemical properties of the four MK homologues investigated in this work.

Property	Ranking
clogP	MK-4 (8.86) > MK-3 (7.52) > MK-2 (5.67) > MK-1 (3.83)
Ability to disrupt a phospholipid monolayer based on increase in monolayer area between 30 and 35 mN/m	MK-4 > MK-2 > MK-1 ~ MK-3
MK headgroup location relative to the midplane	MK-1 ~ MK-2 ~ MK-3 ~ MK-4
Longest average distance from CT to naphthoquinone headgroup	MK-4 > MK-3 > MK-2 > MK-1
Ability of CT to be within 0.6 nm of the naphthoquinone headgroup	MK-1 > MK-2 > MK-4 > MK-3

## Data Availability

The manuscript will be deposited on the NSF repository site. Additional data has been provided in Supplemental Information.

## Supplementary Materials

The following are available online at https://www.mdpi.com/article/10.3390/ijms22189755/s1.
