# Supplementary material for: Interactions of Truncated Menaquinones in Lipid Monolayers and Bilayers
Source: Int J Mol Sci. 2021 Sep 9;22(18):9755. doi: 10.3390/ijms22189755 (PMC8470443; doi:10.3390/ijms22189755)
Supplement: Supplementary file 1 [file ijms-22-09755-s001.zip › Supplementray Information_14012021_CV.pdf]

Article

Interactions of Truncated Menaquinones in Lipid Monolayers and Bilayers

Cameron Van Cleave [1], Jordan T. Koehn [1], Caroline Simões Pereira [2], Allison A. Haase [1], Benjamin J. Peters [1], Seth W. Croslow [3], Kyle G. McLaughlin [3], Katarina R. Werst [1], Audra L. Goach [3], Dean C. Crick [4,5], Guilherme Menegon Arantes [2] and Debbie C. Crans [1,4,*]

[1] Department of Chemistry, Colorado State University, Fort Collins, CO 80523, USA; camvc@colostate.edu (C.V.C.); jordan_koehn@med.unc.edu (J.T.K.); Allison.Haase@colostate.edu (A.A.H.); ben.peters252@gmail.com (B.J.P.); katarina.werst@gmail.com (K.R.W.)

[2] Department of Biochemistry, Institutio de Química, Universidade de São Paulo, Av. Prof. Lineu Prestes 748, 05508-900 São Paulo, SP, Brazil; caroline013@gmail.com (C.S.P.); garantes@iq.usp.br (G.M.A.)

[3] Department of Chemistry, Monmouth College, Monmouth, IL 61462, USA; SCROSLOW@monmouthcollege.edu (S.W.C.); kmclaughlin@mcw.edu (K.G.M.); ASOSTARECZ@monmouthcollege.edu (A.L.G.)

[4] Cell and Molecular Biology Program, Colorado State University, Fort Collins, CO 80523, USA; dean.crick@colostate.edu

[5] Department of Microbiology, Immunology, and Pathology, Colorado State University, Fort Collins, CO 80523, USA

* Correspondence: Debbie.Crans@colostate.edu; Tel.: +1 (970) 491-7635

Table of Contents

S1. PARTITION COEFFICIENTS 3

Table S1.1 .... **Error! Bookmark not defined.**

S2. HYSTERESIS 4

S2.1. Hysteresis Methods 4

Figure S2.1. 5

S3. VISUALIZATION OF COMPRESSION ISOTHERM TERMINOLOGY 6

Figure S3.1 6

S4. COMPRESSION MODULUS 7

S4.1. Compression Modulus Methods 7

S4.2. Interpretation 7

Figure S4.1 8

Figure S4.2 9

Figure S4.3 10

S5. IDEAL MIXING 11

Figure S5.1. 11

Figure S5.2. 12

S6. NOMENCLATURE AND LABELING 13

Figure S6.1 13

Figure S6.2 13

REFERENCES 13

Scheme 1. PARTITION COEFFICIENTS.

Table S1a. Calculated logP values of truncated MK homologues obtained from molinspiration.com.

Analogue	Structure	Calculated logP
MK-1	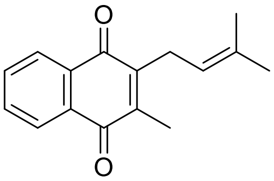	3.83
MK-2	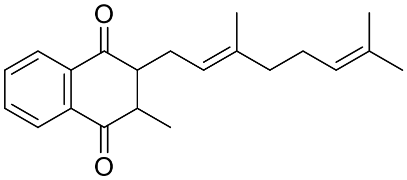	5.67
MK-3	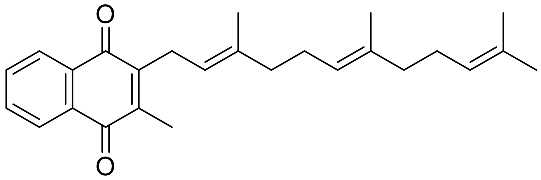	7.52
MK-4	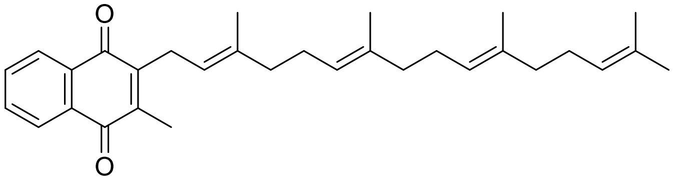	8.86

S2. HYSTERESIS

S2a. Hysteresis Methods

The hysteresis studies of MK films were performed on a Biolin NIMA trough (medium, Teflon) with Teflon block barriers. The injection volumes of 2 mM MK varied based on the ability to generate a surface pressure greater than 1 mN/m. MK-1 required an injection of 400 μL (800 nmol), MK-2 required a 60 μL (120 nmol) injection, and both MK-3 and MK-4 required a 40 μL (80 nmol) injection. As with the compression isotherms, films were allowed to equilibrate for 15 minutes before hysteresis. Films were compressed at a speed of 10 mm/min until a surface pressure between 6 and 8 mN/m. Compression was paused for one second, then expansion proceeded at a speed of 10 mm/min until a surface pressure of 1 mN/m was achieved. This compression/expansion cycle was repeated until the trough ran out of area for compression.

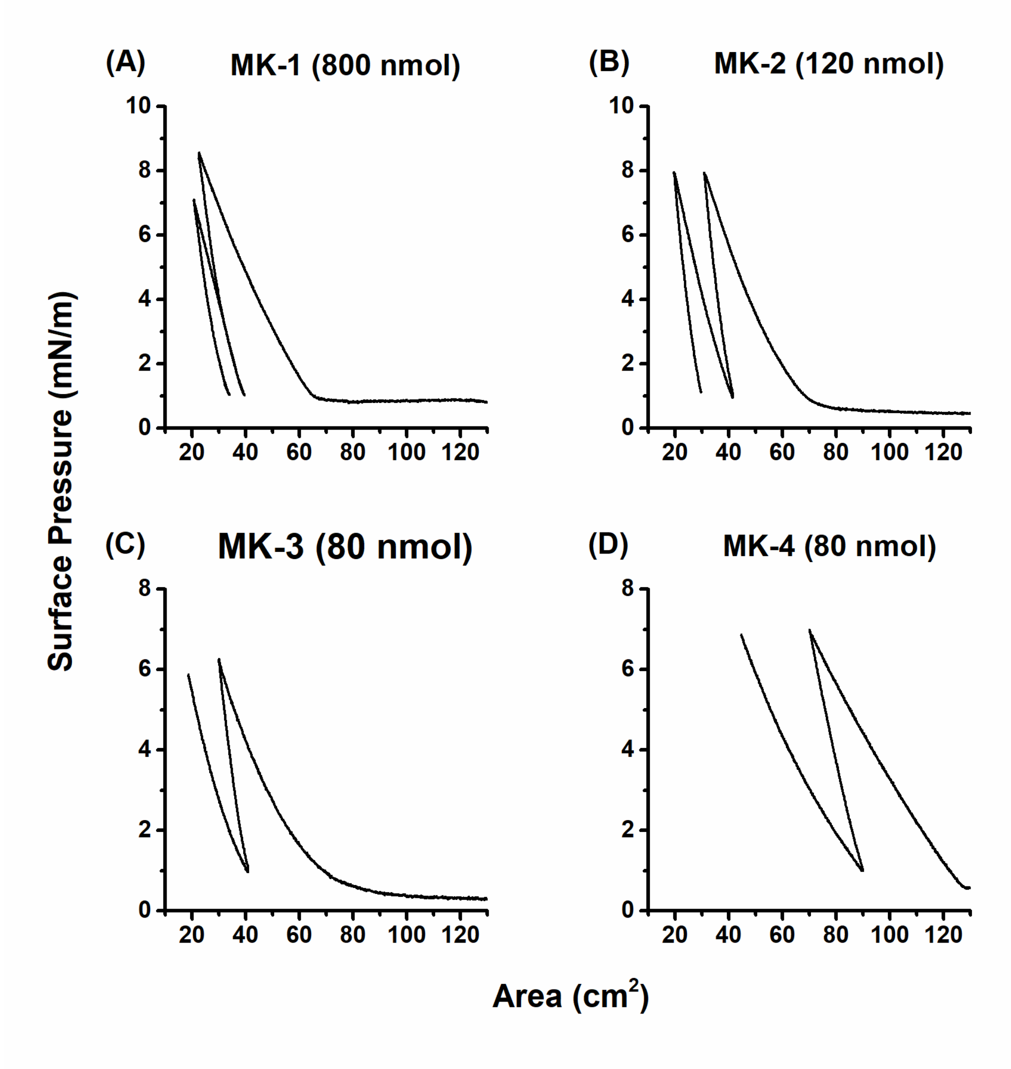

Figure S2a. Hysteresis of pure MK films as a function of surface pressure (mN/m) vs film area (cm²). Panels are representative of (**A**) 800 nmol of MK-1, (**B**) 120 nmol of MK-2, (**C**) 80 nmol of MK-3, and (**D**) 80 nmol of MK-4.

S3. VISUALIZATION OF COMPRESSION ISOTHERM TERMINOLOGY

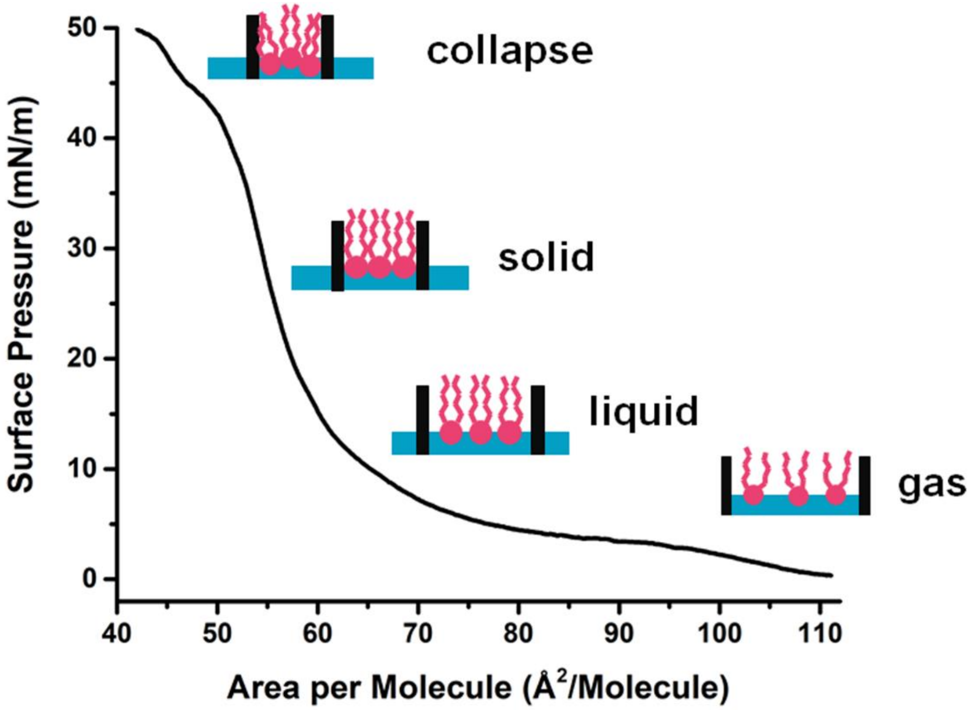

Figure S3a. A representative compression isotherm demonstrating and visualizing the different behavioral phases that are observed and described in Langmuir monolayer studies.

S4. COMPRESSION MODULUS

S4a Compression Modulus Methods

The compression modulus of each average was calculated according to Equation 3, where C_s^{-1} is the compression modulus, A is the area per molecule (Å^2), and π is the surface pressure.

$$C_S^{-1} = -A(\frac{d\pi}{dA})_T \quad \text{(S1)}$$

The first derivative of the surface pressure with respect to the area at a constant temperature was calculated in Origin 2021 and smoothed with a second degree polynomial Savitsky-Golay function (500 points per window). The first derivative was then multiplied by the negative of the area and graphed versus surface pressure in Origin 2021.

S4b Interpretation

The calculated compression modulus showed phase transitions and changes in monolayer compressibility (Figure S4a). The maximum compression modulus (highest point of the curve) may be used to indicate the state of the film (i.e. liquid or solid). These states are thought of as a range as opposed to one indicative value.[1] For example, a maximum compression modulus between 50 and 100 mN/m is indicative of a liquid condensed film. The values obtained in Figure S4.1 mixed phospholipid:MK films are either liquid or liquid-expanded. A general trend for DPPC films mixed with MK is that the maximum compression modulus decreased with increasing MK homologue concentration, indicating a more fluid film. Likewise, increasing MK concentration suppressed the gas-liquid phase transition peak between 0 and 10 mN/m. The DPPE:MK films all demonstrated decrease in maximum compression modulus as the molar fraction of MK increased, similar to DPPC. Despite errors (Figures S4b and S4c), the overall trend of decreased compression modulus with increased MK fraction is clear.

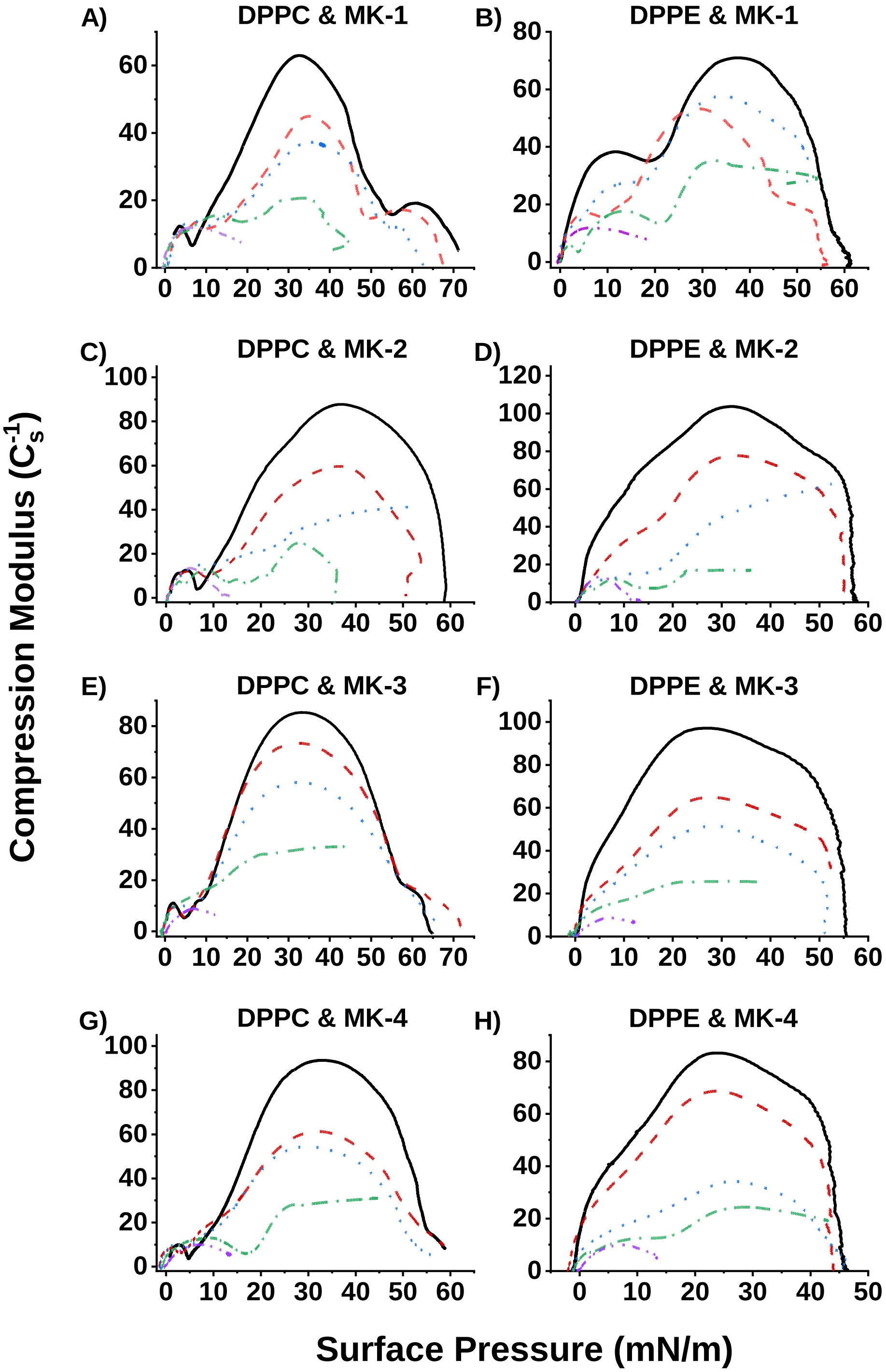

Figure S4a Compression modulus (C_S^{-1}) versus surface pressure (mN/m) of DPPC (left column) and DPPE (right column) and phospholipid:MK films. Panels **A**) and **B**) are MK-1, **C**) and **D**) are MK-2, **E**) and **F**) are MK-3, and **G**) and **H**) are MK-4. Solid black curves represent pure lipid, red dashed curves represent 75:25 phospholipid:MK, blue dotted curves represent 50:50 phospholipid:MK, green dash-dot curves represent 25:75 phospholipid:MK, and purple dash-dot-dot curves represent pure MK.

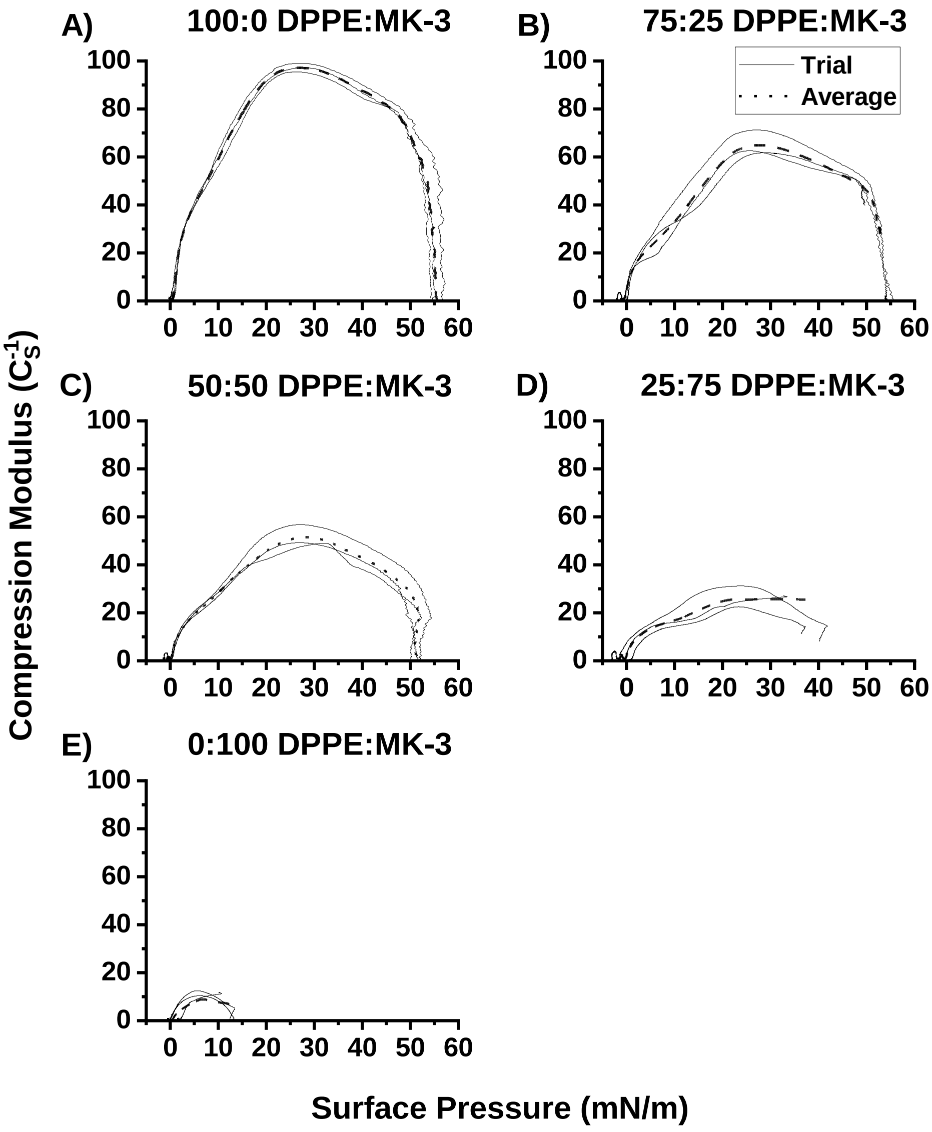

Figure S4b Representative compression modulus versus surface pressure plots demonstrating experimental trials versus calculated averages of (**A**) DPPE, (**B**) 75:25 DPPE:MK-3, (**C**) 50:50 DPPE:MK-3, (**D**) 25:75 DPPE:MK-3, and (**E**) MK-3. These plots demonstrate that the average curve is a decent representation of compression modulus data.

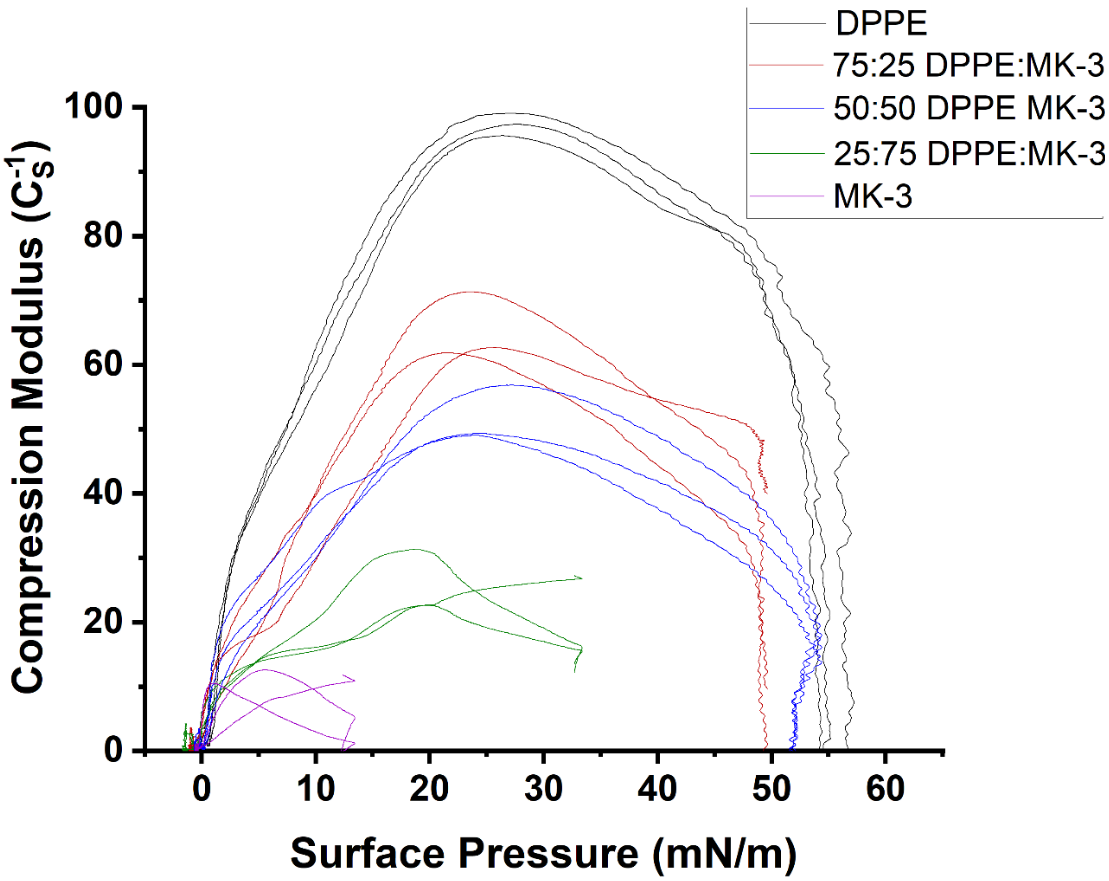

Figure S4c. A representative compression modulus versus surface pressure graph in which all experimental trials of the DPPE:MK-3 monolayers were transformed into compression modulus and plotted versus surface pressure. This demonstrates that while there may be large error, an overall trend of increased compressibility (decreased compression modulus) is identifiable.

S5. IDEAL MIXING

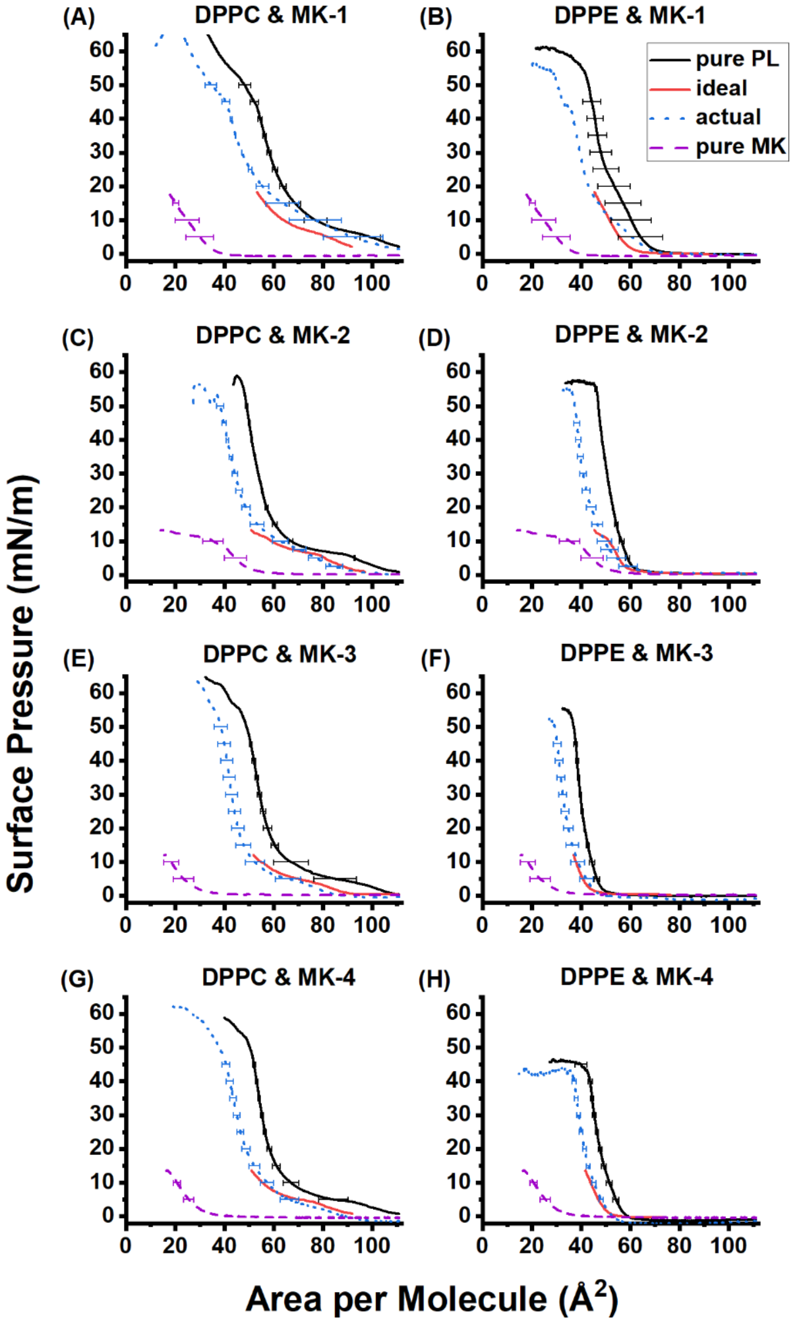

Figure S5a Ideal mixing of 75:25 phospholipid:MK films compared to experimental data. DPPC films are in the left column. DPPE films are in the right column. (**A**) and (**B**) show MK-1 mixed films, (**C**) and (**D**) show MK-2 mixed films, (**E**) and (**F**) show MK-3 mixed films, and (**G**) and (**H**) show MK-4 films. Solid black curves are pure phospholipid monolayers. Blue dotted curves represent experimental 75:25 phospholipid:MK films. Solid red curves represent calculated ideal mixed films. Purple dash-dot-dot curves represent pure MK films. Error bars are the standard deviation of triplicate measurements.

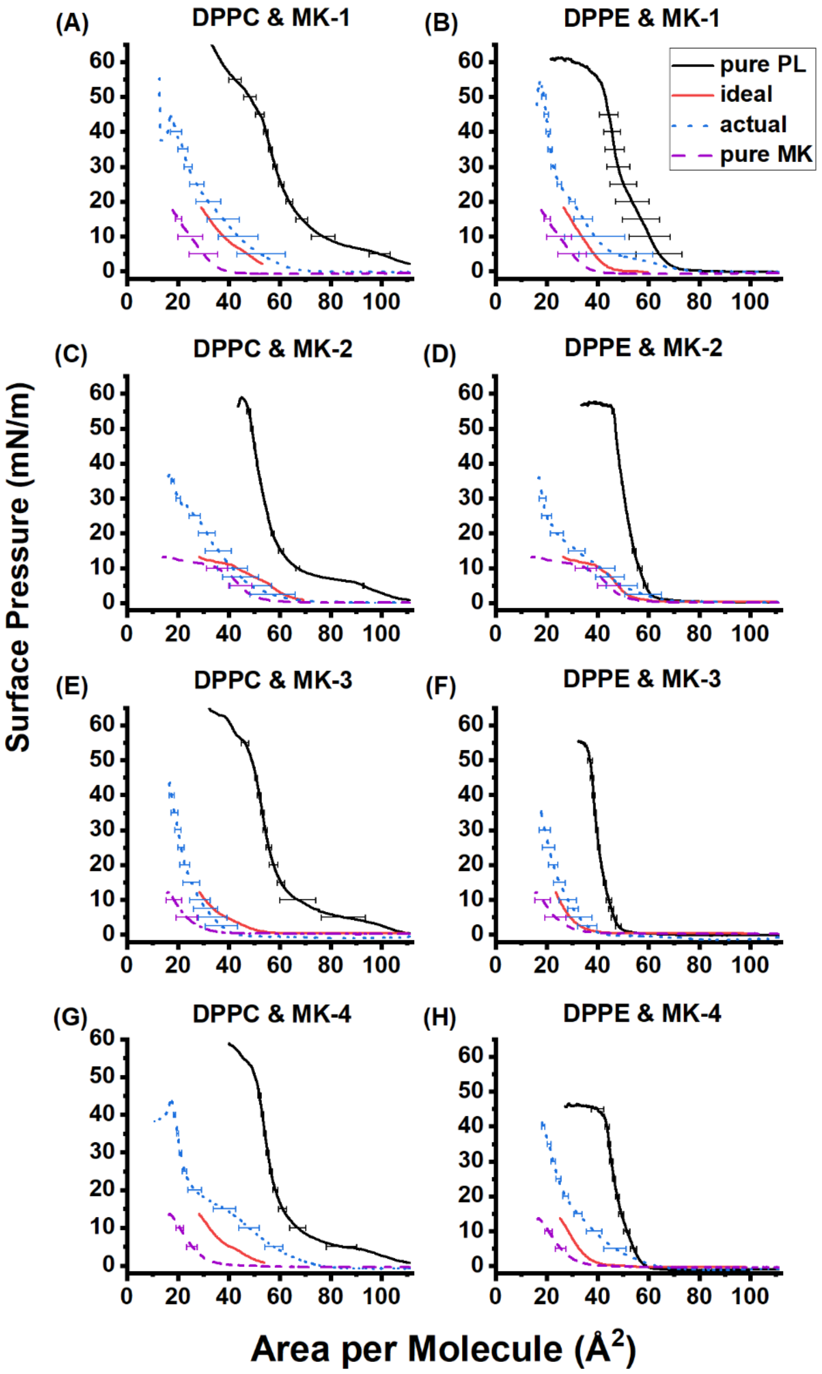

Figure S5b Ideal mixing of 25:75 phospholipid:MK films compared to experimental data. DPPC films are in the left column. DPPE films are in the right column. (**A**) and (**B**) show MK-1 mixed films, (**C**) and (**D**) show MK-2 mixed films, (**E**) and (**F**) show MK-3 mixed films, and (**G**) and (**H**) show MK-4 films. Solid black curves are pure phospholipid monolayers. Blue dotted curves represent experimental 50:50 phospholipid:MK-*n* films. Solid red curves represent calculated ideal mixed films. Purple dash-dot-dot curves represent pure MK films. Error bars are the standard deviation of triplicate measurements.

S6. NOMENCLATURE AND LABELING

The IUPAC name of MK-4 is 2-methyl-3-(3,7,11,15-tetramethylhexadeca-2,6,10,14-tetraenyl)naphthalene-1,4-dione. The labeling scheme is provided below in Figure S6.1. This differs from the labeling scheme in the main manuscript as we used the UQ scheme (Figure S6.2) for easier comparison.

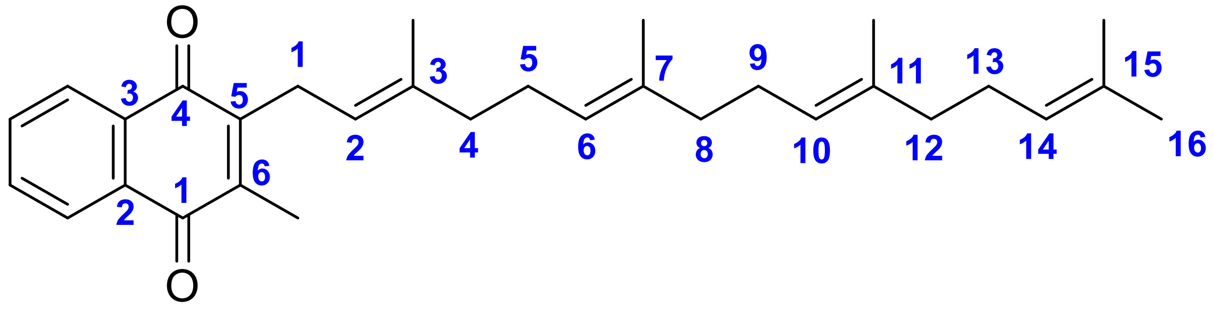

Figure S6a IUPAC labeling of MK-4.

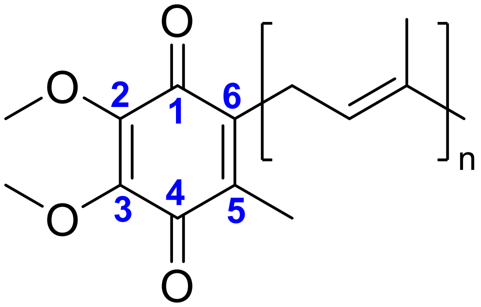

Figure S6b IUPAC labeling of UQ molecules.

REFERENCES

1. Patterson, M.; Vogel, H. J.; Prenner, E. J., Biophysical characterization of monofilm model sytems composed of selected tear film phospholipids. *Biochim. Biophys. Acta Biomembr.* **2016,** *1858*, 403-414.
